# Risk factors associated with nursing-sensitive adverse events in older hospitalised patients: A retrospective chart review

**DOI:** 10.1016/j.ijnsa.2026.100527

**Published:** 2026-04-02

**Authors:** Anna Connolly, Anthony Staines, Anne Matthews, Maria Unbeck, Kasia Bail, Marcia Kirwan

**Affiliations:** a School of Nursing, Psychotherapy and Community Health, Dublin City University, Dublin, Ireland; b School of Health and Welfare, Dalarna University, Falun, Sweden; c Department of Clinical Sciences, Danderyd Hospital, Karolinska Institutet, Stockholm, Sweden; d Centre for Ageing Research and Translation, University of Canberra, and Synergy Nursing and Midwifery Research Centre ACT Health Directorate, Canberra, Australia

**Keywords:** Patient safety, Adverse event, Risk factors, Nursing, Older adult patients

## Abstract

**Background:**

Approximately 10% of hospitalised patients experience adverse events. Older patients are particularly vulnerable to developing nursing-sensitive adverse events such as pneumonia, urinary tract infections, pressure ulcers and delirium. However, various predictors which further increase their risk of acquiring such adverse events are not well understood.

**Objectives:**

To identify factors associated with nursing-sensitive adverse events in patients aged 65 and over in one acute Irish hospital and to determine the relationship between a nursing-sensitive adverse event, a patient’s discharge destination and in-hospital mortality.

**Design:**

A retrospective study using healthcare chart data.

**Methods:**

A cohort of 1000 admissions of inpatients aged 65 and over who were discharged from a single university, tertiary hospital in Ireland in 2022 were included. A two-stage retrospective chart review was conducted on each of the 1000 admissions to extract data pertaining to their hospitalisation and to identify the presence of pneumonia, urinary tract infections, pressure ulcers and delirium. Univariate analysis was used to screen 13 collected variables to identify those with significant relationships with the nursing-sensitive adverse events. Variables with a p value of <0.25 were included in the multivariate analysis which was used to identify significant risk factors for any nursing-sensitive adverse event and each of the studied events in isolation.

**Results:**

A statistically significant association between medical speciality, an increase in age, length of stay, female sex, the number of diagnoses and procedures and a patient’s admission situation and the nursing-sensitive adverse events investigated in this study was identified. Furthermore, time from first presentation to admission and number of ward moves were also associated with nursing-sensitive adverse events but yielded some contradictory findings. A significant association between in-hospital mortality and pressure ulcers was also identified. Patients who experienced nursing-sensitive adverse events were more likely to be discharged with home care packages or to be discharged to nursing homes, other hospitals, rehabilitation facilities or destinations within the ‘other’ category than be discharged home independently.

**Conclusions:**

This study offers valuable insights for the prevention of nursing-sensitive adverse events. Investment in strategies that are focused on medical patients, older patients, patients with multiple co-morbidities and those with longer lengths of stay is required to reduce nursing-sensitive adverse events. Further exploration of the association between waiting times from presentation to admission and ward moves and nursing-sensitive adverse events given that these factors may be potentially impacted by key nursing-modifiable interventions.

**What is already known about the topic?**

• Older patients are increasingly vulnerable to experiencing hospital-acquired adverse events.
• Length of stay, age and frailty are well-established predictors of in-hospital adverse events.

Alt-text: Unlabelled box dummy alt text

**What this paper adds?**

• This builds on existing research exploring the predictors of nursing-sensitive adverse events in older patients by measuring the impact of additional hospital and patient-level factors.
• Medical speciality, an increase in age, length of stay, the number of diagnoses and procedures, female sex and a patient’s admission situation were significantly associated with the nursing-sensitive adverse events investigated in this study. Time from presentation to admission and number of ward moves were also associated with nursing-sensitive adverse events. Pressure ulcers and in-hospital mortality were significantly associated and patients with nursing-sensitive adverse events were more likely to be discharged with further care requirements.
• This study provides an insight into potential clinical and policy implications that may reduce nursing-sensitive adverse events in older patients and improve patient safety and healthcare quality.

Alt-text: Unlabelled box dummy alt text

## 1 Introduction

An adverse event is an incident resulting in patient harm that is healthcare-associated rather than arising from underlying disease or injury (World Health Organisation, 2009). Older patients are particularly vulnerable to adverse events due to their complex care needs (Gupta et al., 2025) with approximately 6–60 % of older patients affected (World Health Organisation, 2024a). They require basic but fundamental nursing care to manage pre-existing conditions in addition to their acute presenting conditions however, when the demand for nursing care exceeds supply, care is prioritised according to clinical requirements to the detriment of fundamental cares (Bail and Grealish, 2016). Pneumonia, urinary tract infections, pressure ulcers and delirium commonly occur in older patients and contribute to functional and cognitive decline. They are measurable indicators of the ‘Failure to Maintain’ conceptual framework which indicates rationed nursing care for older patients (Bail and Grealish, 2016). Therefore, these nursing-sensitive adverse events have been explored in this study. Nurse shortages, inadequate staffing and patient acuity contribute to prioritisation of certain nursing tasks over others (Gong et al., 2025). Consequently, essential nursing tasks are frequently missed, resulting in nursing-sensitive adverse events (He et al., 2025). Nursing-sensitive adverse events, or nurse-sensitive outcomes, are affected by nursing care processes, however nursing is not exclusively responsible for them (National Quality Forum, 2004). System-level and organisational factors such as resource constraints, workflow disruptions, inefficient procedures and processes, inadequate or absent policies and regulations and economic pressures may also contribute to their occurrence (World Health Organization, 2023).

Sex, age, length of stay, intensive care unit admissions (Ahmed et al., 2015) and patient complexity, often underpinned by co-morbidities, older age and healthcare utilisation (Schaink et al., 2012), have been associated with adverse events. More specifically, length of stay, Charlson index scores, operation length and intensive care unit admission are common predictors of pneumonia (Mukhtar et al., 2025) which affects approximately 5.8–7.9 % of older patients (Burton et al., 2016; Connolly et al., 2025). Older patients are particularly vulnerable to urinary tract infections (Gajdács et al., 2021), which occur in approximately 5.2 % of admissions (Connolly et al., 2025), due to age-related deterioration of immune system responses, exposure to healthcare-associated pathogens and an increasing number of comorbidities however, those with a history of urinary tract infections are at high-risk of re-occurrence (Caljouw et al., 2011; Rodriguez-Mañas, 2020). Sex, age, admission method and consciousness status are associated with pressure ulcers (Han et al., 2018), which occur in 8.4 % of hospitalised adults (Li et al., 2020). Although often considered reversible, delirium, which occurs in 10–32 % of admissions (Connolly et al., 2025; Fuchs et al., 2020), can result in long-lasting cognitive decline (Fong et al., 2009). According to Ormseth et al. (2023), non-modifiable delirium risk factors include dementia, age, functional impairment, multiple co-morbidities, and male sex, among others. Modifiable risk factors include immobilisation, medications, co-existing illnesses, anaemia, dehydration and poor nutritional status, metabolic derangement, surgery, environment, pain, emotional distress and sleep deprivation. The nursing-modifiable nature of some of the precipitating factors associated with adverse events, including those investigated through this study, are potential targets for prevention.

Pneumonia, urinary tract infections, pressure ulcers and delirium can potentially predict patient outcomes. Delirium and pressure ulcers are associated with prolonged hospitalisation, an increased likelihood of death within six months and discharge with further care needs (Mudge et al., 2019). Delirium is an independent predictor of length of stay, mortality, nursing home placement and further functional and cognitive decline (Fong et al., 2009). Urinary tract infections and pneumonia are associated with higher rates of in-hospital mortality, prolonged hospitalisation and increased healthcare costs (Gidey et al., 2023). These events can impact a patient’s recovery and result in increased healthcare costs with approximately 15 % of total health expenditure spent on managing patient harm consequences in high-income countries (World Health Organisation, 2024a). In Ireland, the estimated cost of hospital-acquired urinary tract infections, pressure ulcers or pneumonia is €694 per event (Murphy et al., 2021).

Given their clinical and financial burden, understanding the predictors of nursing-sensitive adverse events is key to preventing them, improving healthcare quality and decreasing healthcare expenditure (Dziegielewski et al., 2021). Insights into potential risk factors may help clinicians and healthcare providers develop more targeted interventions (Caljouw et al., 2011; Liu et al., 2024; Mei et al., 2023; Mukhtar et al., 2025). The rapid ageing of the Irish and global population suggests that health systems worldwide need to prepare to meet the care demands associated with this demographic change (World Health Organisation, 2025). Therefore, understanding the factors associated with nursing-sensitive adverse events in older patients is important in an Irish and global context. Although the factors contributing to adverse events in long-term care settings have been investigated (McGrane et al., 2024), few studies have explored the factors associated with pneumonia, urinary tract infections, pressure ulcers and delirium in older patients in acute Irish hospital settings. Furthermore, although administrative data often under-report nursing-sensitive adverse events (Connolly et al., 2025), many studies rely on them to identify risk factors (Georgiev et al., 2025; Hassan et al., 2010; Mukhtar et al., 2025; Toye et al., 2019). Chart review has shown to be a more valid method for identifying adverse events. (Connolly et al., 2024). Therefore, the use of a large retrospective chart review to identify rates of nursing-sensitive adverse events and nursing-modifiable risk factors offers a potentially more accurate analysis of the impact of these factors.

Considering this knowledge gap, the use of chart review as the data collection method and the potentially predictive nature of these conditions in relation to patient outcomes, this study aimed to investigate the factors associated with pneumonia, urinary tract infections, pressure ulcers and delirium in patients aged 65 and over in an acute Irish hospital setting. This research also aimed to determine the relationship between nursing-sensitive adverse events, a patient’s discharge destination and in-hospital mortality.

## 2 Methods

### 2.1 Study design, setting and participants

This study is situated within a wider project which aimed to demonstrate the potential for safer hospital care and reduced healthcare costs associated with older patients in acute hospitals in Ireland through accurate reporting of nursing-sensitive adverse events using hospital administrative data. Further information is available at https://www.dcu.ie/snpch/cost2care.

Retrospective chart review data was collected for a cohort of 1000 admissions of inpatients aged 65 and over who were discharged from a single university, tertiary hospital in Ireland with over 800 beds and 25,000 inpatient discharges in 2022. Irrespective of their discharge destination, patients discharged from inpatient adult wards, including intensive care units between the 1st of January 2022 and the 31st of December 2022 were included. In line with previous research by Mudge et al. (2019), only patients admitted for 72 hours or longer were eligible for inclusion to limit adverse events to those that were hospital-acquired. A total of 6575 admissions met these criteria. A simple simulation run in R using various adverse event rates indicated that a sample of 400 admissions provided an acceptably precise estimate. Larger numbers were required to facilitate subgroup analysis, therefore the sample was stratified into two categories: medical and surgical admissions, consistent with DRG coding. The relative size of the groups, and the expected differences between these categories in terms of prevalence was not known. Therefore, a sample size of approximately 500 per category, resulting in 1000 admissions, was appropriate to give reasonable power to detect between groups and allow for the exploration of the range of likely outcomes (Supplementary Fig. 1).

### 2.2 Variables

#### 2.2.1 Outcomes and definitions

The outcomes measured were pneumonia, urinary tract infections, pressure ulcers, delirium or the presence of any of these during hospitalisation.

##### 2.2.1.1 Delirium

Delirium was confirmed by the term “delirium” and other alternative acceptable terms such as “confusion”, in line with previous literature (Puelle et al., 2015) and as agreed on consultation with participating clinicians.

##### 2.2.1.2 Pressure ulcer

Based on Irish health service guidelines (HSE, 2018), and on consultation with the involved healthcare professionals, documentation of non-blanching redness was accepted as a stage one pressure ulcer. Stage 2, 3 and 4 pressure ulcers were recorded as per the staging documented within the patients’ clinical charts.

##### 2.2.1.3 Pneumonia and UTI

The World Health Organisation (2024b) healthcare-associated infections case definitions were provided by the healthcare professionals involved and used to confirm the presence of pneumonia and urinary tract infections.

The inclusion and exclusion criteria for each of the adverse events are outlined in Supplementary Table 1.

##### 2.2.1.4 Co-production of chart review protocol and data collection instrument

A two-stage retrospective chart review was guided by a study-specific chart review protocol that was co-produced by the researchers, a public and patient involvement representative and hospital representatives. Retrospective chart review expert (MU) provided a review template and advised on variable selection. Furthermore, meetings arranged by the participating hospital’s project coordinator facilitated variable selection discussions with the Director and Assistant Directors of Nursing, an advanced nurse practitioner, a consultant geriatrician, a tissue viability nurse, a clinical nurse specialist and a consultant microbiologist.

##### 2.2.1.5 Overall chart review process

The review took place at the participating hospital between October 2023 and April 2024. Paper-based documentation from nurses, doctors and allied healthcare professionals were reviewed. Laboratory results, imaging records, prescriptions and referral data were accessed via an online information system. Access to the intensive care unit online system was also given. Elixhauser scores were calculated for each patient. A clinical research nurse with previous chart review experience and a PhD student with no chart review experience received training from the chart review expert (MU) and conducted the chart review. A senior research team member monitored the review process.

##### 2.2.1.6 Review stage 1

All admissions were subject to review stage 1. Sex, age, time of admission and discharge, admission situation, discharge destination, number of bed and ward moves, frailty scores, length of stay, hours from presentation to admission, admissions to intensive care unit, speciality and admission route were collected. Ward moves, as outlined by Blay et al. (2017), referred to any transfer of a patient between wards or clinical units such as intensive care units. In line with previous research, the number of bed moves referred to the number of bed changes a patient experienced throughout their admission, including bed transfers between or within wards (Ranasinghe et al., 2017). If one of the predefined adverse events was identified, the admission was forwarded to review stage 2.

##### 2.2.1.7 Review stage 2

Further data pertaining to the identification and treatment of the adverse event, such as severity, antibiotic use, interventions and referrals were collected during review stage 2. The data were entered into the data collection instrument in Excel using either open-text or drop-down menus with preset responses.

### 2.3 Data sources and quantitative variables

#### 2.3.1 Exposure

The exposure variables were speciality (medical/surgical), sex, admission route (elective/emergency), admission situation, hours from presentation to admission, number of ward moves, number of bed moves, age, admission to the intensive care unit, length of stay, number of diagnoses, number of procedures and Elixhauser score. Variable selection was guided by the chart review expert (MU) and the hospital representatives and was informed by previous literature which provided empirical rationale for the included variables. As patient complexity is indicated by age, multimorbidity and healthcare utilisation (Schaink et al., 2012), variables such as age, number of diagnoses and number of procedures were considered important. Previous studies exploring predictors of adverse events indicated that the selected variables were relevant and potentially important (Fong et al., 2009; Han et al., 2018; Itoh et al., 2025; Liu et al., 2024; Mukhtar et al., 2025; Ranasinghe et al., 2017; Webster et al., 2016).

### 2.4 Statistical methods

The methods used to analyse the relationships between the outcome and exposure variables were of an exploratory and associate nature, rather than causal, given that reverse causation cannot be excluded for certain variables.

All variables were screened using univariate analysis to determine variables for inclusion in the multivariate analysis. Binary logistic regression was run on each variable and a p-value cut-off point of <0.25 was used to ensure that important variables were not excluded in the multivariate analysis (Bursac et al., 2008; Chowdhury and Turin, 2020; Zhang, 2016). This process was repeated for each outcome individually, resulting in five models. Variables with a p-value of <0.05 in the multivariate analysis were considered statistically significant. The odds ratio was used to indicate the ratio-change in the odds of a nursing-sensitive adverse event occurring per one-unit increase in the predictor variable.

Univariate analyses were conducted for each nursing-sensitive adverse events to determine their relationship with in-hospital mortality. Similarly, variables with a p-value of <0.25 were included for multivariate analysis and a p-value of <0.05 in the multivariate analysis was considered statistically significant. The relationship between a nursing-sensitive adverse event and a patient’s discharge destination was also explored using multinomial logistic regression. All statistical calculations were performed using IBM SPSS Statistics version 30.

## 3 Results

### 3.1 Participants and descriptive data

A total of 6575 admissions were eligible for review. The sample included 1000 admissions of patients aged 65 and over with a minimum length of stay of 72 hours who were discharged in 2022. Descriptive statistics and the characteristics of the participants are shown in Supplementary Table 2.

### 3.2 Outcomes

A nursing-sensitive adverse event was identified in 231 (23.1 %) admissions. A total of 79, 52, 71 and 100 patients experienced at least one pneumonia, urinary tract infection, pressure ulcer or delirium, respectively. A total of 176 admissions contained one adverse event, 40 admissions contained two adverse events, 14 admissions contained three adverse events, and one admission contained all four. As some patients experienced more than one adverse event, a model assessing the association between the exposure variables and any of the four adverse events was run in addition to four individual event-specific models. Differences were identified between the variables that were associated with any adverse event and those which were associated with the individual adverse events.

### 3.3 Variable selection and model performance

The true positive and false positive rates for each model, demonstrating the number of cases that correctly identified in comparison to the chart review data is outlined in Supplementary Table 3.

#### 3.3.1 Any nursing-sensitive adverse event

A total of 13 potential risk factors were explored in relation to the nursing-sensitive adverse events. A total of 11 variables demonstrated statistical significance (p<0.25) in the univariate analysis. The multivariate analysis indicated that four of these variables were statistically significant.

The full model containing all predictors was statistically significant, X^2^ (14, N = 988) = 272.718, p < 0.001, indicating that the model was able to distinguish between patients who experienced a nursing-sensitive adverse event and patients who did not. The model correctly classified 82.2 % of cases. As shown in Table 1, four of the independent variables significantly contributed to the model. Time from presentation to admission had an odds ratio of 0.981, indicating that patients with longer waiting times were less likely to experience nursing-sensitive adverse events during their hospitalisation. An odds ratio of 1.142 for number of diagnoses demonstrated that patients with more comorbidities were more likely to experience a nursing-sensitive adverse event. Furthermore, an odds ratio of 1.034 demonstrated that patients with a longer length of stay were more likely to experience a nursing-sensitive adverse event. Finally, an odds ratio of 1.041 for age demonstrated that older patients were more likely to experience a nursing-sensitive adverse event. The precision of these odds ratios is indicated by the relatively narrow confidence intervals (Table 1).

**Table 1:** Multivariate logistic regression outputs.

	Outcome variables											
	Any Nursing-Sensitive Adverse Event		Pneumonia		Urinary Tract Infection		Pressure Ulcer			Delirium		
Predictor variables	*p*	OR	*p*	OR	*p*	OR	*p*		OR	*p*		OR
		(95 % CI)		(95 % CI)		(95 % CI)			(95 % CI)			(95 % CI)
Speciality (Reference category = medical)	0.824	1.051	0.048	0.518			0.486		1.260	0.074		1.659
		(0.677–1.632)		(0.269–0.995)					(0.657–2.417)			(0.952–2.891)
Sex (Reference category = male)	-	-	-	-	0.012	2.290	0.133		1.554	-		-
						(1.201–4.365)			(0.874–2.761)			
Admission route (Reference category = elective)	0.982	1.007	-	-	-	-	0.783		0.879	-		-
		(0.573–1.768)							(0.349–2.210)			
Hours from first presentation to admission	0.028	0.981	0.200	0.984	-	-	-		-	0.257		0.988
		(0.965–0.998)		(0.961–1.008)								(0.968–1.009)
Number of ward moves	0.899	1.019	0.437	1.162	0.123	1.446	0.044		0.659	0.043		0.712
		(0.763–1.361)		(0.796–1.695)		(0.905–2.31)			(0.44–0.988)			(0.512–0.99)
Number of bed moves	0.859	1.017	0.919	1.013	0.055	0.712	0.158		1.199	0.100		1.196
		(0.841–1.231)		(0.789–1.301)		(0.504–1.007)			(0.932–1.542)			(0.966–1.481)
Age	0.003	1.041	0.734	0.993	-	-	0.018		1.051	0.003		1.052
		(1.014–1.068)		(0.954–1.034)					(1.008–1.095)			(1.017–1.088)
ICU admission (Reference category = not admitted to ICU)	0.158	1.684	0.866	1.081	-	-	0.100		2.448	0.054		2.283
		(0.817–3.472)		(0.437–2.675)					(0.843–7.106)			(0.985–5.294)
Length of stay	<0.001	1.034	0.028	1.013	<0.001	1.022	0.003		1.017	0.332		1.005
		(1.021–1.047)		(1.001–1.024)		(1.010–1.034)			(1.006–1.028)			(0.995–1.015)
Number of diagnoses	<0.001	1.142	<0.001	1.107	<0.001	1.143	<0.001		1.151	<0.001		1.119
		(1.093–1.192)		(1.049–1.169)		(1.075–1.215)			(1.090–1.216)			(1.067–1.174)
Number of procedures	0.202	1.049	0.040	1.093	0.091	0.913	0.719		0.982	0.104		1.067
		(0.975–1.13)		(1.004–1.189)		(0.821–1.015)			(0.891–1.083)			(0.987–1.153)
Admitted from Home independent (reference category)	0.913		0.355		0.124		0.049			0.241		
Admitted from Home care package	0.745	1.091	0.323	0.646	0.231	0.573	0.023		2.408	0.202		1.544
		(0.645–1.845)		(0.271–1.537)		(0.231–1.424)			(1.131–5.13)			(0.792–3.008)
Admitted from Nursing home	0.546	1.262	0.221	0.275	0.191	0.254	0.090		2.392	0.272		1.663
		(0.593–2.686)		(0.035–2.17)					(0.873–6.554)			(0.670–4.124)
						(0.033–1.978)						
Admitted from Other hospital	0.650	1.159	0.193	1.658	0.975	1.016	0.026		2.614	0.353		0.689
		(0.613–2.189)		(0.774–3.555)		(0.368–2.809)			(1.124–6.081)			(0.314–1.512)
Admitted from Other	0.430	1.404	0.825	1.143	0.072	2.633	0.714		0.679	0.110		2.198
		(0.605–3.26)		(0.35–3.735)		(0.916–7.567)			(0.086–5.391)			(0.836–5.783)
Elixhauser score	-	-	-	-	-	-	-		-	-		-
ICU = Intensive Care Unit												

**Fig. 1:**
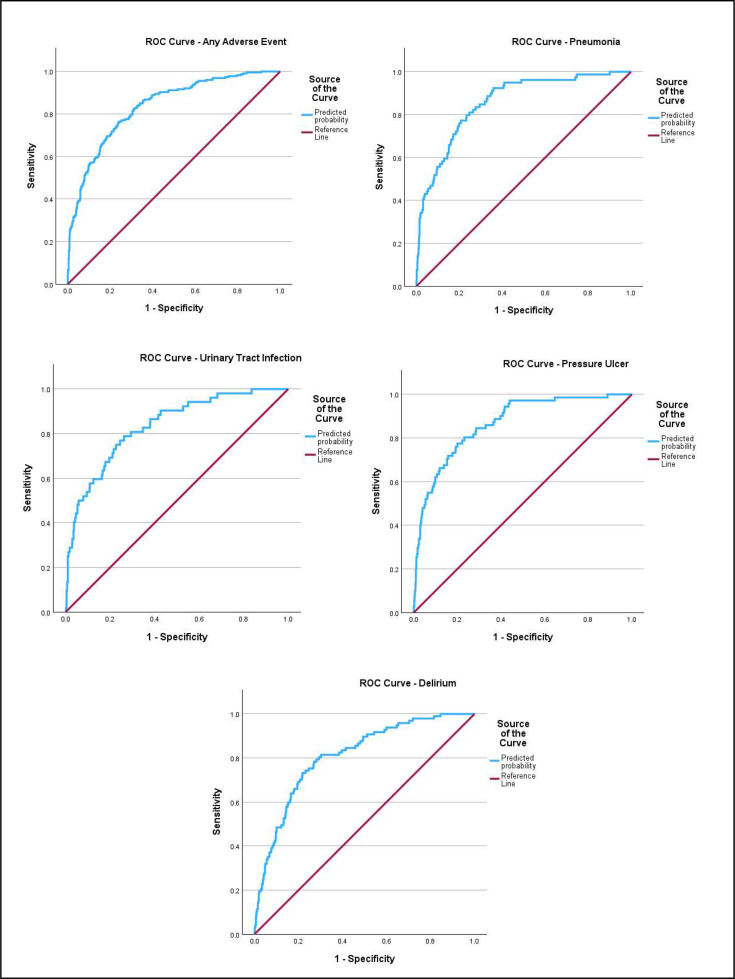
ROC Curves demonstrating the area under the curve for each predictive model. The area under the curve for the model predicting any adverse event was 0.836. The models predicting pneumonia, urinary tract infections, pressure ulcers and delirium had area under the curve values of 0.858, 0.834, 0.867 and 0.811, respectively. Further detail in relation to the area under the curve values, such as confidence intervals, for each logistic regression model is available in supplementary table 4.

The model had an overall accuracy of 82.2 % and demonstrated good fit with a Hosmer-Lemshow of p=0.097 and pseudo R^2^ values between 0.241 and 0.366. An ROC curve (Fig. 1) was generated to assess how well the model could distinguish between patients with and without nursing-sensitive adverse events. The area under the curve (Supplementary Table 4) was 0.836, demonstrating that the model had an 83.6 % chance of correctly classifying patients with and without nursing-sensitive adverse events.

#### 3.3.2 Pneumonia

All 13 potential risk factors were explored in relation to pneumonia. A total of 10 variables demonstrated statistical significance (p<0.25) in the univariate analysis with four of these variables identified as statistically significant in the multivariate analysis.

The full model containing all significant predictors from the univariate analysis was statistically significant, X^2^ (13, N = 988) = 131.955, p < 0.001, indicating that the model was able to distinguish between patients who experienced a pneumonia and patients who did not. The model correctly classified 92.5 % of cases. As shown in Table 1, four of the independent variables made a statistically significant contribution to the model. An odds ratio of 0.518 for speciality demonstrated that surgical patients were less likely than medical patients to experience a pneumonia. An odds ratio of 1.107 for number of diagnoses demonstrated that patients with more comorbidities were more likely to experience a pneumonia. Furthermore, an odds ratio of 1.093 for a number of procedures demonstrated that patients with more procedures were more likely to experience a pneumonia. Finally, an odds ratio of 1.013 for length of stay, demonstrated that patients with a longer length of stay were more likely to develop a pneumonia. The narrow confidence intervals for the odds ratio for number of diagnoses, number of procedures and length of stay demonstrate the precision of the effect however, the relatively broad confidence intervals for speciality indicate a level of uncertainty in relation to the effect (Table 1).

The model had an overall accuracy of 92.5 %% and demonstrated good fit with a Hosmer-Lemshow of p=0.121 and pseudo R^2^ values between 0.125 and 0.293. An ROC curve (Fig. 1) was generated and the area under the curve (Supplementary Table 4) was 0.858, demonstrating that the model had an 85.8 % chance of correctly classifying patients with and without a pneumonia.

#### 3.3.3 Urinary tract infection

All 13 potential risk factors were explored in relation to urinary tract infections. A total of 7 variables demonstrated statistical significance (p<0.25) in the univariate analysis. The multivariate analysis indicated that three of these variables were statistically significant.

The full model containing all significant predictors from the univariate analysis was statistically significant, X^2^ (10, N = 994) = 82.621, p < 0.001, indicating that the model was able to distinguish between patients with and without urinary tract infections. The model correctly classified 94.8 % of cases. As shown in Table 1, three variables made a statistically significant contribution to the model. Sex had an odds ratio of 2.290, demonstrating that females were more likely than males to develop urinary tract infections. An odds ratio of 1.143 suggested that patients with more diagnoses were more likely to experience a urinary tract infection. Finally, an odds ratio of 1.022 for length of stay, demonstrated that patients with a longer length of stay were more likely to experience a urinary tract infection. The relatively narrow confidence intervals for length of stay and number of diagnoses indicate the precision of the effect size. However, although the association between sex and urinary tract infection is significant, the confidence intervals were broad (Table 1).

The model had an overall accuracy of 94.8 % and demonstrated good fit with a Hosmer-Lemshow of p=0.774 and pseudo R^2^ values between 0.08 and 0.237. An ROC curve (Fig. 1) was generated and the area under the curve (Supplementary Table 4) was 0.834, demonstrating that the model had an 83.4 % chance of correctly classifying patients with and without urinary tract infections.

#### 3.3.4 Pressure ulcer

The same 13 potential risk factors were investigated in relation to pressure ulcers. A total of 11 variables demonstrated statistical significance (p<0.25) in the univariate analysis. The multivariate analysis indicated that five of these variables were statistically significant (Table 1).

The full model containing all significant predictors from the univariate analysis was statistically significant, X^2^ (14, N = 990) = 136.253, p < 0.001, indicating that the model was able to distinguish between patients with and without pressure ulcers. The model correctly classified 93.2 % of cases. As shown in Table 1, five variables made a statistically significant contribution to the model.

An odds ratio of 0.659 for number of ward moves demonstrated that patients with less ward moves were more likely to experience a pressure ulcer. Furthermore, the odds ratio of 1.151 for number of diagnoses demonstrated that patients with more comorbidities were more likely to experience a pressure ulcer. Admission situation was also identified as a predictor of pressure ulcers with patients with homecare packages and those who were transferred from other hospitals more likely to develop pressure ulcers than those living independently given the odds ratios of 2.408 and 2.614, respectively. An odds ratio of 1.017 for length of stay demonstrated that patients with a longer length of stay were more likely to experience a pressure ulcer. An odds ratio of 0.659 for number of ward moves demonstrated that patients with more ward moves were less likely to experience a pressure ulcer. Finally, age was a significant predictor with an odds ratio of 1.051, demonstrating that the chance of developing a pressure ulcer increases with age. The confidence intervals for each of these odds ratios, as outlined in Table 1, demonstrate the precision of the effect size of each of these variables.

The model had an overall accuracy of 93.2 % and demonstrated good fit with a Hosmer-Lemshow of p=0.158 and pseudo R^2^ values between 0.129 and 0.319. An ROC curve (Fig. 1) was generated and the area under the curve (Supplementary Table 4) was 0.867, demonstrating that the model had an 86.7 % chance of correctly classifying patients with and without pressure ulcers.

#### 3.3.5 Delirium

The 13 potential risk factors were also investigated in relation to delirium. A total of 10 variables demonstrated statistical significance (p<0.25) in the univariate analysis. The multivariate analysis indicated that three of these variables were statistically significant.

The full model containing all significant predictors from the univariate analysis was statistically significant, X^2^ (13, N = 988) = 113.785, p < 0.001, indicating that the model was able to distinguish between patients with and without delirium. The model correctly classified 90 % of cases. As shown in Table 1, three of the variables made a statistically significant contribution to the model. Patients with more comorbidities were more likely to experience delirium with an odds ratio of 1.119. An odds ratio of 0.712 for number of ward moves demonstrated that patients with more ward moves were less likely to experience a delirium. Finally, age was a significant predictor with an odds ratio of 1.052, demonstrating that the chance of developing delirium increases with age. The confidence intervals which demonstrate the precision of the effect size of the association between these variables and delirium is outlined in Table 1.

The model had an overall accuracy of 90.2 % and demonstrated good fit with a Hosmer-Lemshow of p=0.553 and pseudo R^2^ values between 0.109 and 0.230. An ROC curve (Fig. 1) was generated and the area under the curve (Supplementary Table 4) was 0.811, demonstrating that the model had an 81.1 % chance of correctly classifying patients with and without delirium.

### 3.4 Relationship between the presence of a nursing-sensitive adverse event and in-hospital mortality and discharge destination

#### 3.4.1 In-hospital mortality

Binary logistic regression indicated that in-hospital mortality was 4.954 times more likely to occur in patients with any nursing-sensitive adverse event (Table 2). Univariate analysis on each nursing-sensitive adverse event individually demonstrated that patients complicated with pneumonia, pressure ulcers and delirium were significantly (p<0.25) more likely to experience in-hospital mortality. Although urinary tract infections were not statistically significant, due to their clinical significance, they were included in the model. Multivariate analysis results indicated that pressure ulcers, with an odds ratio of 6.864, were the only statistically significant contributor to in-hospital mortality (Table 2). The confidence intervals were relatively wide, therefore indicating that although the association is significant, the effect size is uncertain. The full model containing all significant predictors from the univariate analysis was statistically significant, X^2^ (4, N = 1000) = 28.145, p < 0.001, indicating that the model was able to distinguish between patients who died in hospital and patients who did not.

**Table 2:** Binary and multivariate logistic regression demonstrating the association between nursing-sensitive adverse events and in-hospital mortality.

Binary logistic regression model to test the association between any nursing-sensitive adverse event and in-hospital mortality		
Outcome variable	*p*	OR (95 %)
Any Nursing-sensitive Adverse Event	<0.001	4.954 (2.555–9.604)
Multivariate logistic regression model to test the association between each of the studied nursing-sensitive adverse events and in-hospital mortality		
Outcome variables	*p*	OR (95 % CI)
Pneumonia	0.102	2.127 (0.862–5.25)
Urinary Tract Infection	0.867	0.894 (0.240–3.324)
Pressure Ulcer	<0.001	6.864 (3.177–14.83)
Delirium	0.730	1.177 (0.465–2.982)

The model had an overall accuracy of 96.2 % and demonstrated good fit with a Hosmer-Lemshow of p=0.504 and pseudo R^2^ values between 0.028 and 0.101. An ROC curve (Supplementary Fig. 2) was generated and the area under the curve (Supplementary Table 5) was 0.7, demonstrating that the model had a 70 % chance of correctly classifying patients who died in hospital.

#### 3.4.2 Discharge destination

The multinomial logistic regression suggested that patients with a nursing-sensitive adverse event were significantly more likely than those without to be discharged with a homecare package (OR=2.295, p≤0.001), to a nursing home (OR=4.164, p≤0.001), to another hospital (OR=4.404, p≤0.001) or rehabilitation facility (OR=4.648, p≤0.001) or to fall within the ‘other’ category (OR=6.996, p≤0.001) than to be discharged home independently (Table 3). The associations identified are statistically significant however, the confidence intervals are relatively broad (Table 3).

**Table 3:** Multinomial logistic regression demonstrating association between nursing-sensitive adverse event occurrence and discharge destination.

Discharge Destination		Std. Error	*p*	OR (95 % CI)
Homecare package	Intercept	0.103	<0.001	
	[Any NSAE Multinomial=Yes]	0.219	<0.001	2.295 (1.494–3.524)
	[Any NSAE Multinomial=No]	-	-	-
Nursing Home	Intercept	0.177	<0.001	
	[Any NSAE Multinomial=Yes]	0.299	<0.001	4.164 (2.316–7.490)
	[Any NSAE Multinomial= No]	-	-	-
Other hospital	Intercept	0.168	<0.001	
	[Any NSAE Multinomial= Yes]	0.282	<0.001	4.404 (2.533–7.655)
	[Any NSAE Multinomial= No]	-	-	-
Rehabilitation facility	Intercept	0.143	<0.001	
	[Any NSAE Multinomial= Yes]	0.243	<0.001	4.648 (2.888–7.481)
	[Any NSAE Multinomial= No]	-	-	-
Other	Intercept	0.213	<0.001	
	[Any NSAE Multinomial= Yes]	0.314	<0.001	6.996 (3.784–12.933)
	[Any NSAE Multinomial= No]	-	-	-

Note: The reference category is home independent

NSAE = Nursing-sensitive adverse event

A multinomial logistic regression was conducted to determine any association between the four nursing-sensitive adverse events and a patient’s discharge destination, with all events adjusted for within the same model (Table 4). Patients with pneumonia were significantly more likely than those without to be discharged to another hospital (OR=2.791, p≤0.009) or to fall within the ‘other’ category (OR=3.512, p≤0.003) than to be discharged home independently. Patients with urinary tract infections had an increased likelihood of being discharged to a rehabilitation facility (OR=5.920, p≤0.001) in comparison to being discharged home independently. Pressure ulcers were associated with an increased odds of being discharged with a homecare package (OR=6.105, p≤0.001) or being discharged to a nursing home (OR=7.582, p≤0.001), other hospital (OR=9.774, p≤0.001), rehabilitation facility (OR=5.85, p≤0.001) or to a destination within the ‘other’ category (OR=16.246, p≤0.001) than to be discharged home independently. Patients with delirium were significantly more likely than those without to be discharged with a homecare package (OR=2.697, p≤0.002) or to be discharged to a nursing home (OR=3.945, p≤0.001), other hospital (OR=3.604, p≤0.001) or rehabilitation facility (OR=4.435, p≤0.001). These associations were statistically significant however, the confidence intervals for some variables are relatively broad.

**Table 4:** Multinomial logistic regression demonstrating association between each individual nursing-sensitive adverse event and discharge destination.

Discharge Destination		Std. Error	*p*	OR (95 % CI)
Homecare package	Intercept	0.101	<0.001	
	[Pneumonia Multinomial=Yes]	0.466	0.212	0.559 (0.224–1.394)
	[UTI Multinomial= Yes]	0.490	0.670	1.232 (0.471–3.222)
	[Pressure Ulcer Multinomial= Yes]	0.418	<0.001	6.105 (2.690–13.854)
	[Delirium Multinomial= Yes]	0.314	0.002	2.697 (1.457–4.994)
Nursing Home	Intercept	0.168	<0.001	
	[Pneumonia Multinomial= Yes]	0.492	0.445	1.456 (0.555–3.817)
	[UTI Multinomial= Yes]	0.589	0.219	2.062 (0.65–6.542)
	[Pressure Ulcer Multinomial= Yes]	0.509	<0.001	7.582 (2.796 -20.565)
	[Delirium Multinomial= Yes]	0.400	<0.001	3.945 (1.803–8.633)
Other hospital	Intercept	0.163	<0.001	
	[Pneumonia Multinomial= Yes]	0.394	0.009	2.791 (1.288–6.048)
	[UTI Multinomial= Yes]	0.598	0.454	1.564 (0.485–5.049)
	[Pressure Ulcer Multinomial= Yes]	0.466	<0.001	9.774 (3.919–24.374)
	[Delirium Multinomial= Yes]	0.385	<0.001	3.604 (1.696–7.657)
Rehabilitation facility	Intercept	0.140	<0.001	
	[Pneumonia Multinomial= Yes]	0.384	0.141	1.761 (0.829–3.742)
	[UTI Multinomial= Yes]	0.382	<0.001	5.920 (2.801–12.51)
	[Pressure Ulcer Multinomial= Yes]	0.467	<0.001	5.85 (2.344–14.602)
	[Delirium Multinomial= Yes]	0.330	<0.001	4.435 (2.321–8.477)
Other	Intercept	0.192	<0.001	
	[Pneumonia Multinomial= Yes]	0.423	0.003	3.512 (1.533–8.047)
	[UTI Multinomial= Yes]	0.623	0.303	1.901 (0.561–6.444)
	[Pressure Ulcer Multinomial= Yes]	0.469	<0.001	16.246 (6.475–40.763)
	[Delirium Multinomial= Yes]	0.483	0.133	2.067 (0.802–5.326)

UTI = Urinary Tract Infection

## 4 Discussion

This research identified important factors associated with nursing-sensitive adverse events in patients aged 65 and over in an acute Irish hospital. A significant associative relationship was identified between speciality, time from presentation to admission, age, number of diagnoses, length of stay, number of procedures, sex, admission situation and number of ward moves and the nursing-sensitive adverse events. Patient complexity, as indicated by age, multimorbidity and number of procedures (Schaink et al., 2012), was significantly associated with adverse events. This research also identified an association between in-hospital mortality and pressure ulcers. Furthermore, patients with nursing-sensitive adverse events were more likely to be discharged with additional care needs as rather than home independently.

In this study, surgical patients were less likely than medical patients to develop pneumonia. In addition to a higher incidence of hospital-acquired pneumonia in medical departments in comparison to surgical departments (Sopena et al., 2014), an association between medical admissions and adverse events such as pressure ulcers, falls and aspiration pneumonia has previously been demonstrated (Adamuz et al., 2020). As medical patients tend to be older patients with chronic diseases and other co-morbidities (Adamuz et al., 2018), they may have poorer health than surgical patients, and may more vulnerable to pneumonia. Exploration of nursing-modifiable interventions tailored to medical patients’ needs may result in more positive patient outcomes.

In this study, increasing age was associated with an increased likelihood of any nursing-sensitive adverse event, and pressure ulcers and delirium, in particular. An association between greater mean age and an increased likelihood of adverse events has also been identified by San Jose-Saras et al. (2022). The findings relating to pressure ulcers are supported by previous research which found that the likelihood of a pressure ulcer increased 1.04 times per year of age (Han et al., 2018). These findings suggest that efforts to reduce nursing-sensitive adverse events should focus on older and therefore, more vulnerable patients.

Length of stay is another widely recognised risk factor for adverse events (Hassan et al., 2010; Mukhtar et al., 2025; Rosman et al., 2015). Longer admissions are often inevitable in patients with underlying co-morbidities who require complex procedures that increase their likelihood of adverse events (Mukhtar et al., 2025). In line with previous literature identifying an increased risk of infection and pressure ulcers for each overnight hospital stay (Hauck and Zhao, 2011), this study demonstrated an association between increased length of stay and an increased likelihood of pneumonia, urinary tract infections and pressure ulcers or any nursing-sensitive adverse event. Therefore, patients with increased lengths of stay should be prioritised to reduce their vulnerability to nursing-sensitive adverse events. However, the bidirectional relationship between length of stay and adverse events must be acknowledged as adverse events may result from and contribute to prolonged hospitalisation. Reverse causation cannot be excluded, therefore the associative, rather than causal, nature of this relationship is emphasised as adverse events may significantly prolong length of stay (Fan et al., 2024; Hoogervorst-Schilp et al., 2015; Tessier et al., 2019). Given the potential for an adverse event-related increased length of stay to result in poor-patient outcomes and increased healthcare costs, it is essential that adverse events are prevented.

Similar to figures from Violan et al. (2014), 94.9 % of admissions in this study were affected by multimorbidity. A patient’s number of diagnoses was associated with an increased likelihood of any nursing-sensitive adverse event and each event when considered in the individual models. These findings reflect findings of a 1.4-fold increased risk of adverse events in patients with two or more comorbidities (Nghiem et al., 2022). Hospitals should focus their efforts on prioritising particularly vulnerable patients, such as those with multiple comorbidities. Furthermore, additional research exploring a transition from single-disease specific interventions to bundles of interventions to prevent multiple nursing-sensitive adverse events is required.

The likelihood of pneumonia was associated with an increased number of procedures. This finding is unsurprising given that patients with longer lengths of stay who require more complex procedures inevitably have an increased likelihood of adverse events (Mukhtar et al., 2025). This finding suggests that hospitals should focus pneumonia prevention strategies on post-procedural periods as patients appear to be particularly vulnerable at this stage of their treatment. Post-procedural patient care is a nursing-modifiable factor that should be further explored in terms of reducing nursing-sensitive adverse events in older patients.

In this study, patients receiving homecare and those transferred from other hospitals were more likely to develop pressure ulcers. Inter-hospital transfers have been associated with poor patient outcomes and increased risks of in-hospital mortality (Song et al., 2024). These findings suggest that specific strategies and interventions targeted towards patients who are transferred from other hospitals and those receiving help with daily living may be beneficial for reducing nursing-sensitive adverse events. The care given to transferred patients is a nursing-modifiable factor which should be considered in relation to the development of prevention strategies.

Although there is limited research exploring sex differences in patient harm (World Health Organisation, 2024a), research suggests that women are disproportionately affected (Institute for Health Metrics and Evaluation, 2024). In line with previous research (Al Qahtani et al., 2024), this study identified that females were more likely than males to develop urinary tract infections. In terms of clinical implications, this finding demonstrates that sex-specific strategies may be required in order to prevent urinary tract infections.

Although many factors in this study are supported by previous literature, some findings are inconsistent with previous research. Prolonged emergency department stays and increased waiting times to admission due to bed unavailability, increased care demands and slow patient flow has been associated with adverse events (do Nascimento Rocha et al., 2021). Conversely, longer waiting times to admission in this study was significantly associated with a reduction in nursing-sensitive adverse events. Triage processes in Irish hospitals ensure that the sickest patients are identified and treated first (Health Service Executive, 2025), therefore patients who are less vulnerable to adverse events, may experience longer wait times. The clinical implication of this suggests that patients with acute conditions and an increased risk of adverse events should be prioritised and admitted sooner than those who are healthier and less susceptible to adverse events. However, this is a strictly exploratory hypothesis that requires further investigation. This contradictory finding must be considered with caution given the associative, rather than causal, relationship. Further investigation is required given the weakness of the association which indicated a 1.9 % reduced chance of any nursing-sensitive adverse event.

A significant association between ward moves and delirium and pressure ulcers was also identified. However, in contrast to previous research, an increased number of ward moves was associated with a reduced risk of delirium. Again, as reverse causation cannot be excluded, it is important to consider that the relationship identified between ward moves and adverse events is associative rather than causal. Previous research demonstrates that ward transfers are associated with an increased likelihood of falls, wound infections, and delirium (Blay et al., 2017; Ranasinghe et al., 2017; Toye et al., 2019; Webster et al., 2016). However, complex clinical journeys have been associated with ward transfers, with patients with co-occurring clinical needs requiring ward transfers for more specialised treatment. (Mendelsohn et al., 2023). The reduced risk of delirium and pressure ulcers in relation to ward moves may reflect the movement of patients to speciality services tailored to their needs, therefore mitigating their risk of delirium or pressure ulcers. As ward move locations were not recorded, their reflection of movement for more specialised care is a strictly exploratory hypothesis rather than a definitive mechanism to explain this contradictory finding. Furthermore, non-clinical ward moves, where patients are temporarily transferred to different wards, are a short-term solution to overcrowding in hospitals (George and Wilkinson, 2016). Therefore, patients who are healthier, and less vulnerable to adverse events, may experience more ward moves when bed capacity is limited. It is also possible that as patients become healthier, and less susceptible to adverse events, they may experience more transfers to less specialised wards. Again, these interpretations are strictly exploratory hypotheses that have not been investigated. Therefore, further research is required to explore these associations. A more in-depth understanding of the impact of factors, such as communication and coordination during ward transfers may also yield interesting findings for mitigating adverse events.

Similar to previous findings (Nghiem et al., 2022; San Jose-Saras et al., 2022), patients with at least one nursing-sensitive adverse event in this study were more likely to die in hospital than those who did not. Furthermore, and in line with previous research (Pang et al., 2025; Song et al., 2019), pressure ulcers in this study were associated with a higher risk of mortality. Although increased severity of pressure ulcers has been associated with an increased risk of in-hospital mortality (Labeau et al., 2021), this study did not explore the effect of pressure ulcer severity on in-hospital mortality and therefore stage 1, 2, 3 and 4 pressure ulcers were grouped together in the analysis. Nevertheless, this highlights the impact of nursing-sensitive adverse events on patient outcomes and emphasises the importance of developing strategies to prevent their occurrence and the subsequent further decline in health outcomes for patients.

Patients with nursing-sensitive adverse events were more likely to be discharged with additional care needs as opposed to being discharged home independently. These findings have significant implications in relation to the availability and financial impact of such resources for health systems and patients. These findings, in addition to the projected increase in the demand for long-term care and home support services in Ireland (Walsh and Kakoulidou, 2025) have significant policy implications as adequate funding, staffing and infrastructure will be required to deliver these additional services. In order to reduce the economic impact and the burden to the patient of requiring additional care on discharge, the prevention of nursing-sensitive adverse events should be prioritised in terms of a patient safety agenda.

As highlighted by the World Health Organization (2024a), strategies targeting infections and pressure ulcers are essential in terms of reducing patient harm and the economic burden of patient safety incidents globally. Due to the increased risk of harm for vulnerable populations with complex care needs indicated by their increasing age, comorbidity burden and the various procedures they require, patient safety issues must be addressed (World Health Organisation, 2024a). Nursing-sensitive adverse events can significantly increase the cost and duration of hospital stays. Substantial investments in strategies to prevent nursing-sensitive adverse events may reduce the economic and human cost of patient safety incidents for both health systems and patients (Tessier et al., 2019). Hospitals should focus their efforts on vulnerable populations such as medical patients, older patients, patients with multiple co morbidities and those with longer lengths of stay.

Strategies focused on incorporating continuous risk assessments throughout patients’ hospitalisations may be useful for preventing adverse events given that patients with longer lengths of stay and those who have had procedures appear to be at an increased risk. Furthermore, targeted screening of medical patients and those transferred from other hospitals may also be beneficial. Strategies that ensure additional monitoring of patients who are deemed at risk of adverse events may also reduce the impact of adverse events. However, such interventions require additional nursing demand, therefore investment in strategies focused on nurse workforce planning and sufficient nurse-patient ratios is essential. Understanding the factors associated with adverse events may also inform strategies for optimising resource allocation and ensure that resources are distributed to high-risk patients.

The increasing rate of population ageing demonstrates that health systems worldwide need to be prepared to meet the care demands associated with this demographic shift (World Health Organisation, 2025). Therefore, the results of this study are relevant in an international context, offering insights into the aspects of care for older patients which require immediate attention to reduce patient safety incidents. Furthermore, as the need to validate administrative datasets globally has previously been identified (Connolly et al., 2024), this study offers researchers internationally an insight into the benefits of using a chart review to identify adverse events rates and their associated risk factors, as opposed to relying on administrative data which are often inaccurate (Connolly et al., 2025).

Furthermore, research is required to develop and evaluate the effectiveness of care bundles aimed at preventing multiple nursing-sensitive adverse events as previous research has demonstrated their effectiveness in preventing hospital-acquired pressure ulcers (Demir and Karadag, 2025). Care bundles that combine the effective preventative nursing interventions outlined by Bail and Grealish (2016) could potentially prevent nursing-sensitive adverse events.

### 4.1 Strengths and limitations

A strength of this study was the inclusion of a large university teaching hospital with a large population of secondary care and tertiary care patients, as well as some national speciality patients, which provided a sample that was representative of a diverse range of patient complexities. Furthermore, the participating hospital is reflective of healthcare settings with large patient populations both nationally and internationally facing similar challenges such as patient acuity, resource and staff shortages and the management of both complex and routine cases. Additionally, the retrospective chart review component is a strength of this study as rather than relying on administrative datasets with potentially inaccurate rates of nursing-sensitive adverse events (Connolly et al., 2025), the rates in this study were identified by chart reviewers who adhered to a structured protocol and received training from a chart review expert.

Although the large number of reviewed charts is a methodological strength of this study, the relatively rare outcome occurrence may limit the predictive ability and strength of the power of the study. A limitation of retrospective chart data, which must be considered in relation to these findings, includes the underreporting of delirium (Titlestad et al., 2024) however, all efforts were made to capture both confirmed and unconfirmed delirium. The relatively weak evidence of association between the predictor variables and the nursing-sensitive adverse events somewhat limits these findings. Nevertheless, the findings yielded statistical significance, therefore indicating that the variables investigated may contribute to nursing-sensitive adverse events. Furthermore, the univariate analyses demonstrate that many variables were considered statistically significant predictors of nursing-sensitive adverse events to some extent, with p-values of <0.25. Further research is warranted to explore the weight of contribution that these factors have to the development of nursing-sensitive adverse events in older hospitalised patients.

The inclusion of only one hospital limits the generalisability of these findings. Including additional hospital models may have increased the applicability of these findings to other healthcare settings however, this was not possible due the limited labour resources available. A methodological consideration and limitation of this study is the potential collinearity among the variables indicating clinical complexity such as number of diagnoses and procedures and Elixhauser comorbidity score. The conceptual overlap of these variables and their potential correlation may reduce the precision of the multivariate logistic regression model (Ranganathan et al., 2017). Furthermore, another limitation is the potential interrelation between the nursing-sensitive adverse events. Although separate event-specific mutlivariable models were used, as some patients experienced more than one adverse event, these outcomes may be interdependent and therefore limit the precision of the models.

## 5 Conclusion

This study demonstrates the association between various risk factors and nursing-sensitive adverse events. The findings offer valuable insights into reducing nursing-sensitive adverse events and their associated healthcare costs. Given the impact of nursing-sensitive adverse events on patient outcomes, their prevention is imperative. Investment in strategies focused on vulnerable populations such as medical patients, older patients, patients with multiple co-morbidities and those with longer lengths of stay is required. A focus on risk assessments, continuous monitoring of patients and allocation of resources and nursing care to those at most risk may be useful. However, nurse workforce planning and adequate nurse-patient ratio policies are pivotal to implementing such strategies. The development and implementation of care bundles aimed at preventing multiple adverse events may also yield more positive patient outcomes. A focus on nursing-modifiable factors such as post-procedural care and care tailored to the needs of medical patients and transfer patients is also required to reduce nursing-sensitive adverse events. Additionally, funding and workforce planning policies are required to meet the care demands of the ageing population who are particularly vulnerable to nursing-sensitive adverse events.

## Funding

This research was funded by the 10.13039/100010414Health Research Board under the grant ILP-HSR-2022–009.

## Ethical approval

Ethical approval was obtained from the Dublin City University Research Ethics Committee and the participating hospital’s Institutional Review Board.

## CRediT authorship contribution statement

**Anna Connolly:** Writing – review & editing, Writing – original draft, Methodology, Investigation, Formal analysis, Data curation, Conceptualization. **Anthony Staines:** Writing – review & editing, Formal analysis. **Anne Matthews:** Writing – review & editing, Supervision. **Maria Unbeck:** Writing – review & editing. **Kasia Bail:** Writing – review & editing. **Marcia Kirwan:** Writing – review & editing, Supervision, Resources, Project administration, Methodology, Funding acquisition, Data curation, Conceptualization.

## Declaration of competing interest

The authors declare the following financial interests/personal relationships which may be considered as potential competing interests:

Anna Connolly reports financial support was provided by 10.13039/100010414Health Research Board. If there are other authors, they declare that they have no known competing financial interests or personal relationships that could have appeared to influence the work reported in this paper.

## Appendix Supplementary materials

Image, application 1

Image, application 2

Image, application 3

Image, application 4

Image, application 5

Image, application 6

Image, application 7
