# Supplementary figures and images for: Risk factors associated with nursing-sensitive adverse events in older hospitalised patients: A retrospective chart review
Source: Int J Nurs Stud Adv. 2026 Apr 2;10:100527. doi: 10.1016/j.ijnsa.2026.100527 (PMC13087750; doi:10.1016/j.ijnsa.2026.100527)

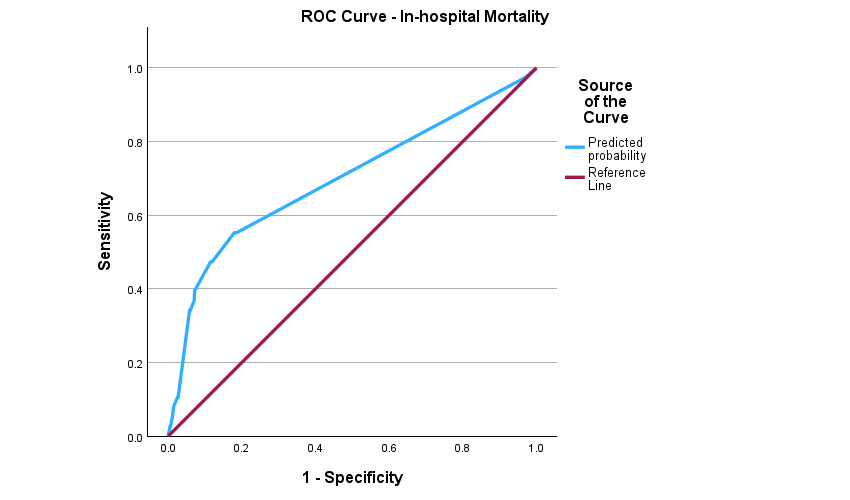


Supplementary Figure 2: ROC Curve for in-hospital mortality

Supplement: Supplementary file 2 [file mmc2.docx]
