# Supplementary material for: Risk factors associated with nursing-sensitive adverse events in older hospitalised patients: A retrospective chart review
Source: Int J Nurs Stud Adv. 2026 Apr 2;10:100527. doi: 10.1016/j.ijnsa.2026.100527 (PMC13087750; doi:10.1016/j.ijnsa.2026.100527)
Supplement: Supplementary file 4 [file mmc4.docx]

Supplementary Table 2

Descriptive statistics

| **Descriptive Statistics (Scale variables)** | | | | | | | | |
| --- | --- | --- | --- | --- | --- | --- | --- | --- |
|  | | Median | Minimum | Maximum | | Mean | Std. Deviation |  |
| **Time from first presentation to admission (hours)** | | 7.23 | 0 | 98.62 | | 12.03 | 14.30 |  |
| **Age discharge** | | 76 | 65 | 98 | | 77.22 | 7.61 |  |
| **Length of stay** | | 9 | 3 | 200 | | 17.26 | 22.94 |  |
| **Elixhauser score** | | .00 | -7 | 17 | | 2.68 | 4.76 |  |
| **Number of diagnoses** | | 6 | 1 | 30 | | 7.22 | 5.24 |  |
| **Total number of procedures** | | 4 | 0 | 20 | | 4.53 | 3.52 |  |
| **Descriptive statistics (categorical variables)** | | | | | | | | |
|  |  | | | | N (%) | | | |
| **Speciality** | Medical | | | | 505 (50.5%) | | | |
|  | Surgical | | | | 495 (49.5%) | | | |
| **Sex** | Male | | | | 534 (53.4%) | | | |
|  | Female | | | | 466 (46.6%) | | | |
| **Admission route** | Emergency | | | | 769 (76.9%) | | | |
|  | Elective | | | | 231 (23.1%) | | | |
| **Admission situation** | Home, independent | | | | 638 (63.8%) | | | |
|  | Home, with homecare package | | | | 155 (15.5%) | | | |
|  | Nursing home | | | | 51 (5.1%) | | | |
|  | Other hospital | | | | 110 (11%) | | | |
|  | Rehab* | | | | 10 (1%) | | | |
|  | Other* | | | | 35 (35%) | | | |
| **Discharge Destination** | Home, independent | | | | 580 (58%) | | | |
|  | Home, with homecare package | | | | 156 (15.6%) | | | |
|  | Nursing home | | | | 56 (5.6%) | | | |
|  | Other hospital | | | | 64 (6.4%) | | | |
|  | Rehab | | | | 93 (9.3%) | | | |
|  | Death** | | | | 38 (3.8%) | | | |
|  | Other** | | | | 10 (1%) | | | |
| **Admitted to ICU** |  | | | | 86 (8.6%) | | | |
| **Age** | 65-74 | | | | 429 (42.9%) | | | |
|  | 75-84 | | | | 362 (36.2%) | | | |
|  | 85+ | | | | 207 (20.7%) | | | |
| **Length of stay** | ≤ 7 days | | | | 387 (38.7%) | | | |
|  | 8-14 days | | | | 284 (28.4%) | | | |
|  | ≥15 days | | | | 327 (32.7%) | | | |
| *For analysis purposes the rehab and other categories were combined.  **For analysis purposes the death and other categories were combined.  ICU = Intensive Care Unit | | | | | | | | |
