# Supplementary material for: Risk factors associated with nursing-sensitive adverse events in older hospitalised patients: A retrospective chart review
Source: Int J Nurs Stud Adv. 2026 Apr 2;10:100527. doi: 10.1016/j.ijnsa.2026.100527 (PMC13087750; doi:10.1016/j.ijnsa.2026.100527)
Supplement: Supplementary file 5 [file mmc5.docx]

Supplementary Table 3: True positive and false positives rates for each model

|  | True positive | False positive |
| --- | --- | --- |
| Any Nursing-sensitive Adverse Event | 226 | 5 |
| Pneumonia | 76 | 3 |
| Urinary Tract Infection | 51 | 1 |
| Pressure Ulcer | 70 | 2 |
| Delirium | 100 | - |
