# Supplementary material for: Risk factors associated with nursing-sensitive adverse events in older hospitalised patients: A retrospective chart review
Source: Int J Nurs Stud Adv. 2026 Apr 2;10:100527. doi: 10.1016/j.ijnsa.2026.100527 (PMC13087750; doi:10.1016/j.ijnsa.2026.100527)
Supplement: Supplementary file 6 [file mmc6.docx]

Supplementary Table 4: Area under the curve (AUC) for each logistic regression model

| Outcome | AUC (95% CI) | Standard error | Asymptotic sig. |
| --- | --- | --- | --- |
| Any nursing-sensitive Adverse Event | 0.836 (0.807–0.865) | 0.015 | 0.000 |
| Pneumonia | 0.858 (0.816–0.899) | 0.021 | 0.000 |
| Urinary Tract Infection | 0.834 (0.779–0.890) | 0.029 | 0.000 |
| Pressure Ulcer | 0.867 (0.826–0.909) | 0.021 | 0.000 |
| Delirium | 0.811 (0.768–0.854) | 0.022 | 0.000 |
