# Supplementary material for: Risk factors associated with nursing-sensitive adverse events in older hospitalised patients: A retrospective chart review
Source: Int J Nurs Stud Adv. 2026 Apr 2;10:100527. doi: 10.1016/j.ijnsa.2026.100527 (PMC13087750; doi:10.1016/j.ijnsa.2026.100527)
Supplement: Supplementary file 7 [file mmc7.docx]

Supplementary Table 5: Area under the curve (AUC) for in-hospital mortality

| Outcome | AUC (95% CI) | Standard error | Asymptotic sig. |
| --- | --- | --- | --- |
| In-hospital mortality | 0.697 (0.596–0.798) | 0.051 | 0.000 |
